# The physical activity at work (PAW) study protocol: a cluster randomised trial of a multicomponent short-break intervention to reduce sitting time and increase physical activity among office workers in Thailand

**DOI:** 10.1186/s12889-020-09427-5

**Published:** 2020-09-01

**Authors:** Cynthia Chen, Anna Valeria Dieterich, Jemima Jia En Koh, Katika Akksilp, Eunice Huiying Tong, Nuttakarn Budtarad, Andre Matthias Müller, Thunyarata Anothaisintawee, Bee Choo Tai, Waranya Rattanavipapong, Wanrudee Isaranuwatchai, Thomas Rouyard, Ryota Nakamura, Falk Müller-Riemenschneider, Yot Teerawattananon

**Affiliations:** 1 Saw Swee Hock School of Public Health, National University of Singapore, Singapore, Singapore; 2 Department of Health, Ministry of Public Health,Health Intervention and Technology Assessment Programme (HITAP), Ministry of Public Health, 6th Floor, 6th Building, Tiwanon Road, Nonthaburi, 11000 Thailand; 3 Hitotsubashi Institute for Advanced Study, Hitotsubashi University, Kunitachi, Japan

**Keywords:** Sedentary behaviour, Physical activity, Productivity, Behaviour change techniques, Quality of life, Non-communicable diseases, Multicomponent intervention, Cost-effectiveness

## Abstract

**Background:**

High levels of sedentary behaviour (SB) are associated with non-communicable diseases. In 2016, the estimated total healthcare expenditure from physical activity (PA) in Thailand added up to $190 million in international dollars. The challenge to reduce SB and increase PA among office workers is more urgent now than ever as Thailand is transforming itself from a predominantly rural country to an increasingly urban one. This study will investigate the effectiveness of a multicomponent short break intervention on the reduction of SB during office hours.

**Methods/design:**

This two-armed Physical Activity at Work (PAW) cluster randomised controlled trial will recruit 360 office workers from 18 offices in the Thailand’s Ministry of Public Health (MOPH). Offices will be randomised to either the intervention group or the control group. The multicomponent intervention is informed by the Social Ecological Model and Behaviour Change Techniques (BCTs) and contains four components: (i) organisational, including heads of the participating divisions leading exercises, sending encouragement text messages and acknowledging efforts; (ii) social, including team movement breaks and team-based incentives; (iii) environmental, including posters to encourage exercise; and (iv) individual components including real-time PA feedback via an individual device. The main intervention component will be a short break intervention. The primary outcome of this study is the sedentary time of office workers. Secondary outcomes include time spent on PA, cardiometabolic outcomes, work productivity, musculoskeletal pain, and quality of life. The study also includes process and economic evaluations from the individual and societal perspective.

**Discussion:**

The study will be the first experimental study in Thailand to investigate the effect of a short-break intervention at the workplace on SBs of office workers and health outcomes. The study will also include a cost-effectiveness analysis to inform investments on short break interventions under the Universal Healthcare Coverage in Thailand, which includes health promotion and disease prevention component.

**Trial registration:**

The PAW study has been registered at the Thai Clinical Trials Registry (TCTR) under the study ID TCTR20200604007. Registered 02 June 2020,

## Background

The negative effects of sedentary behaviour (SB) and a lack of physical activity (PA) on health have been well documented [1–8]. SB is defined as any waking time activity during which one is seated, reclined or lying, having an energy expenditure ≤1.5 metabolic equivalents (METs), while PA is defined as any activity with an energy expenditure > 1.5 METs [9–11]. High levels of SB are associated with a 112% increase in the risk of diabetes, 147% increase in the risk of cardiovascular disease, 90% increase in the risk of cardiovascular mortality and 49% increase in the risk of all-cause mortality [4]. In addition, SB has detrimental associations with fasting glucose, fasting insulin, triglycerides, high-density lipoprotein cholesterol and waist circumference [1].

The negative effects of the behaviour have also been studied in Thailand. According to recent estimates, 6.3% of all-cause mortality in Thailand is due to physical inactivity [12]. The estimated total healthcare costs from physical inactivity in Thailand accounted for $190 million in international dollars (INT$ was calculated using purchasing power parity conversion factors from 2013) [13]. The proportion of individuals not meeting 150 min of moderate-to-vigorous PA in Thailand have increased from 18.5% in 2008 to 19.2% in 2014 [14]. According to the Thai Health Promotion Foundation, Thais spend on average 2 h a day engaging in PA and about 13 h in SB [14].

A long-studied effect of urbanisation is the transition from jobs with more manual work (such as agriculture) to non-manual service jobs that are typically desk-bound [15]. Typical office workers spend most of their SB hours at the office. This is particularly true for computer-based occupations, where employees spend a substantial amount of time in uninterrupted sitting [16–18]. A study found that office-based workers spent up to 75.8% of their working time sitting [19]. Further, breaks between these sitting times were uncommon, with 25% of the total sitting time in bouts of 55 or more minutes [17]. This directly translates to a lower energy expenditure, where such workers expended around ~ 700 kcals/day, compared to individuals whose jobs require some manual labour (~ 2300 kcals/day) [20]. As work-time contributes significantly to the total sedentary time, working hours are an important avenue to address movement behaviours.

It has been suggested that one way to attenuate the negative effects of SB is to increase PA. Studies have illustrated that after adjusting for PA, the negative associations with SB were less pronounced [2, 5]. Physical inactivity represents the non-achievement of PA guidelines, and based on the World Health Organisation global recommendations on PA for health state, adults should do at least 150 min of moderate-intensity aerobic PA throughout the week [21]. Having short PA breaks during working hours can help office workers meet these recommendations.

Short break interventions in the workplace have shown reductions in sedentary time [22]. However, mixed results have been found for their impact on intermediate health outcomes such as calories spent, cholesterol (HDL-C and LDL-C), triglycerides, fasting blood glucose, blood pressure and stress level [22–30]. Importantly, results of a recent randomised trial showed that taking two long breaks (15 min) per workday is less effective than taking shorter breaks (1-2 min) every 30 min [22, 24]. In this study, the long-break group had no change in health outcomes while the short-break group had small-to-moderate declines in total cholesterol (d = − 0.33; *p* = 0.10), triglycerides (d = − 0.38; *p* = 0.06), and fasting blood glucose (d = − 0.29, *p* = 0.01). Even though short breaks every half an hour seems to be more clinically effective, it is less likely to be feasible and scalable in real world practice. Moreover, the above studies were conducted only in high-income countries. No study has investigated the effects of a short-break intervention on the reduction on SB and its impacts on health and productivity outcomes at the workplace in low- and middle-income countries where the majority of metabolic diseases occurs. This study aims to fill this gap.

The study is designed as a two parallel-group cluster randomised superiority trial. The primary outcome assesses sedentary time at the 6th month. The aims of the study are to evaluate (i) the impact of a multicomponent short-break office intervention on minutes spent sedentary during office hours. In addition, the study will also evaluate the effect of the intervention on secondary outcomes such as PA, cardiometabolic risk factors and productivity (ii) the sustainability of the behavioural change; and (iii) the cost-effectiveness of the short-break office intervention. The cost-effectiveness analysis will allow the results to be compared with other trials’ economic evaluations. The results of this study will assist the Thai government in developing health promotion and disease prevention benefit package under the Universal Healthcare Coverage (UHC) scheme. Filling this knowledge gap might also help inform investments on short break interventions in the private sectors.

## Method

### Study design

#### Setting

The study will be located at the Department of Medical Services (DMS), Thailand’s Ministry of Public Health (MOPH) in Nonthaburi Province. Employees are working in central offices with fixed desks. Work tasks comprise mostly computer-based work, but also involve meetings and travelling to meet with officers from other ministries. The offices are all located in the same building on levels 2 to 6.

#### Study design and randomisation

This study is a stratified cluster randomised controlled trial with two arms. We will include at least 18 offices (9 offices per arm) from the DMS, MOPH, forming five strata. These strata are chosen by office sizes (< 15, 15-20, 21-25, 26-34 and ≥ 35 participants). Block randomisation with block sizes of two, four and six will be used to generate the randomised sequence for each stratum. Cluster randomisation by offices minimises the problem of cross-contamination between the intervention arms due to environmental changes in the offices.

#### Blinding

At baseline data collection, participants and data collectors will be blinded to group allocation. Randomisation and allocation of intervention will be performed after the completion of baseline data collection, and participants will be notified via email by researchers not involved in data collection. The allocation sequence will be generated using a computer program which will be created for each stratum. Due to the nature of the intervention, participants are required to know whether they are in the intervention or control group and does not allow for blinding. At follow-up measurements, participants and data collectors will know the treatment allocation. The involvement of research teams from different institutions and countries will allow one team to conduct the randomisation, another to conduct the analysis, and the team from Thailand will implement the trial and perform the data collection. This will ensure that the researchers conducting the analysis will be blinded to the assignment. The analyst will only know the study identifier but not the participant’s name, identifiers, and treatment allocation. Participants’ data (e.g. case record forms, laboratory test, information sheets, and consents) will be stored in a locked cabinet in a researchers’ office. All data will be destroyed by researchers within 5 years after publications. The study protocol has been registered at Thai Clinical Trials Registry (TCTR20200604007) [31]. The study also follows the recommendations of the SPIRIT guidelines. A SPIRIT checklist with references to the relevant page numbers of this protocol is provided (see Additional file 3).

### Participant recruitment

#### Inclusion and exclusion criteria

Office workers with the following criteria are eligible to participate in this trial: 1) have an end date for employment after the study completion date, 2) are legally able to consent, i.e. age 18 years and above, 3) do not have a disability on the upper or lower body that limits their mobility, 4) have a permanent office workstation within one of the clusters included in the trial, 5) works either from their permanent offices or at home at least 3 days a week 6) owns and uses a smartphone compatible with the Fitbit application and 7) are willing to be randomised into one of the two study interventions. Office workers will be excluded from the study if one is away on an extended leave or a personal retreat for more than 2 weeks or is pregnant.

#### Recruitment

While randomisation will be conducted at a cluster level, consent to participate will be sought on a cluster level by leaders, thereafter on the individual level. Office workers will be invited to participate in this study by email. Interested office workers will be screened for eligibility via an online survey. Candidates who meet the inclusion and exclusion criteria will be invited for baseline measurements. Prior to the measurements, study requirements will be explained again and written informed consent will be obtained. Participation will be voluntary, and participants can withdraw at any time without giving any reason. Participants will not face a penalty or consequences of any kind from withdrawing. If participants agree to participate in follow-up assessments after their withdrawal, researchers will conduct these assessments. Departments of MOPH that are not involved in short-break interventions will be considered if there is insufficient participation from DMS. During the recruitment phase, the research team will be in close contact with team leaders of the participating offices and will attend team meetings to facilitate recruitment when needed. To aid with the enrolment, information sessions about the study will be held at DMS.

### Interventions

#### Intervention development approach

The Physical Activity at Work (PAW) intervention development was guided by the Social-Ecological Model, which highlights that behaviours are shaped by various factors on different levels [32, 33]. On the individual level, behaviours can be influenced by attitudes and motivations toward activity. Factors at the social level, such as support from colleagues to be physically active, and at the environmental level, such as conducive space to move can also have a significant influence. Finally, factors at the organisational level (e.g. structures and support in the form of employee encouragement) is likely to play an important role in this study context. Our intervention will address such key factors on each level. The intervention components include different Behaviour Change Techniques (BCTs) to encourage sustained behaviour change. BCTs are active ingredients of the intervention as they are supposed to motivate behavior change [34]. An additional file shows an overview of BCTs and SEM levels that are used in this study (see Additional file 2).

#### Information booklet

Both the control and intervention groups will receive an information booklet about the benefits of PA and “7 easy exercises to an active lifestyle” for the office environment. Creating awareness and educating the public on the health consequences of PA and SB is a common method used in health promotion policies. Thus, the information booklet will contain details on the negative consequences of SB and highlight the benefits of PA. In addition, the booklet containing easy exercises would lower the barrier by providing individuals with the knowledge to elicit personal change.

### Intervention group

#### Individual-level components

##### Fitbit

The intervention group will be given a Fitbit device (Inspire HR) to track their PA throughout the trial. The proper use of the device will be explained in a self-help program booklet. Fitbit is an activity device that uses a tri-axis accelerometer to provide real-time feedback and allows self-monitoring of steps, as well as calories burned, distance covered, active minutes, heart rate, time asleep each day and proportion of hourly activity completed amongst others [35]. It provides feedback through a smartphone app via Bluetooth connection. The device also sets haptic vibration feedback for hourly activity during office hours (9 am to 6 pm) to nudge participants to walk at least 250 steps each hour. Participants will be encouraged to wear the device as frequent as possible to obtain the most accurate results.

##### Lottery-based incentives

Members in the intervention group will be eligible for a weekly performance-based lottery as a form of financial incentive to be physically active. To be eligible, participants will need to complete at least 70% of the recommended number of short breaks (i.e. at least 14 breaks per week out of 20 breaks), with a minimum of 100 steps during each break. Each week, a winner will be randomly selected among eligible participants and will receive 500 THB (US$ 16). Such incentives have been shown to be effective in motivating health-related behaviour change [34]. Adherence to the movement breaks will be measured using the Fitbit devices. Fitbit intraday data will be downloaded, providing minute-by-minute data that will be available through a web application programming interface (API).

#### Physical environment-level components

##### Posters

The intervention group will also be exposed to posters of exercises and stretching, posters with information about the health consequences of SB and PA and posters with details about the PAW study. The posters will be displayed in participants’ offices to encourage them to be physically active. The posters are in an additional file (see Additional file 2).

#### Social-level components

##### Team movement breaks

The social intervention consists of organising short group-based movement breaks, with each cluster forming a team. Each cluster will be encouraged to take four movement breaks of at least 4 min each at 9.30 am, 10.30 am, 2.30 pm and 3.30 pm, within a 60 min time window after the listed timings. An exercise or dance video with music will be played, and participants are encouraged to dance/exercise along with the video. Alarms will signal that it is time for a movement break. If participants work from home, they will be encouraged to join the breaks together online through video conferencing software such as Zoom.

##### Team-based incentives

The weekly winner of the performance-based lottery will receive a bonus of 500 THB (US$ 16) if at least 70% of the teammates also met the recommended target (by completing at least 70% of the weekly recommended short breaks). This bonus is designed to further leverage peer effects by reinforcing accountability and peer support to the team [36, 37]. Participants will be informed about the financial incentives in advance in the information booklet provided to them at the start of the intervention.

#### Organisational-level components

##### Leadership support

Leaders within DMS will encourage participants to take part in the study via Line and Facebook, offering rewards and recognition to participants who win in a weekly lottery (see **lottery-based incentives** above for more details). Line™ is a freeware app for instant communications on electronic devices such as smartphones, tablet computers, and personal computers that is used by 44 million residents in Thailand or 63% of the Thai population [38]. Participants who meet the recommended short break targets will receive recognition for their efforts by email, and each weekly winner of the performance-based lottery will receive their prize from the head of the participating division.

### Outcomes and measurements

#### Data collection

**Fig. 1:**
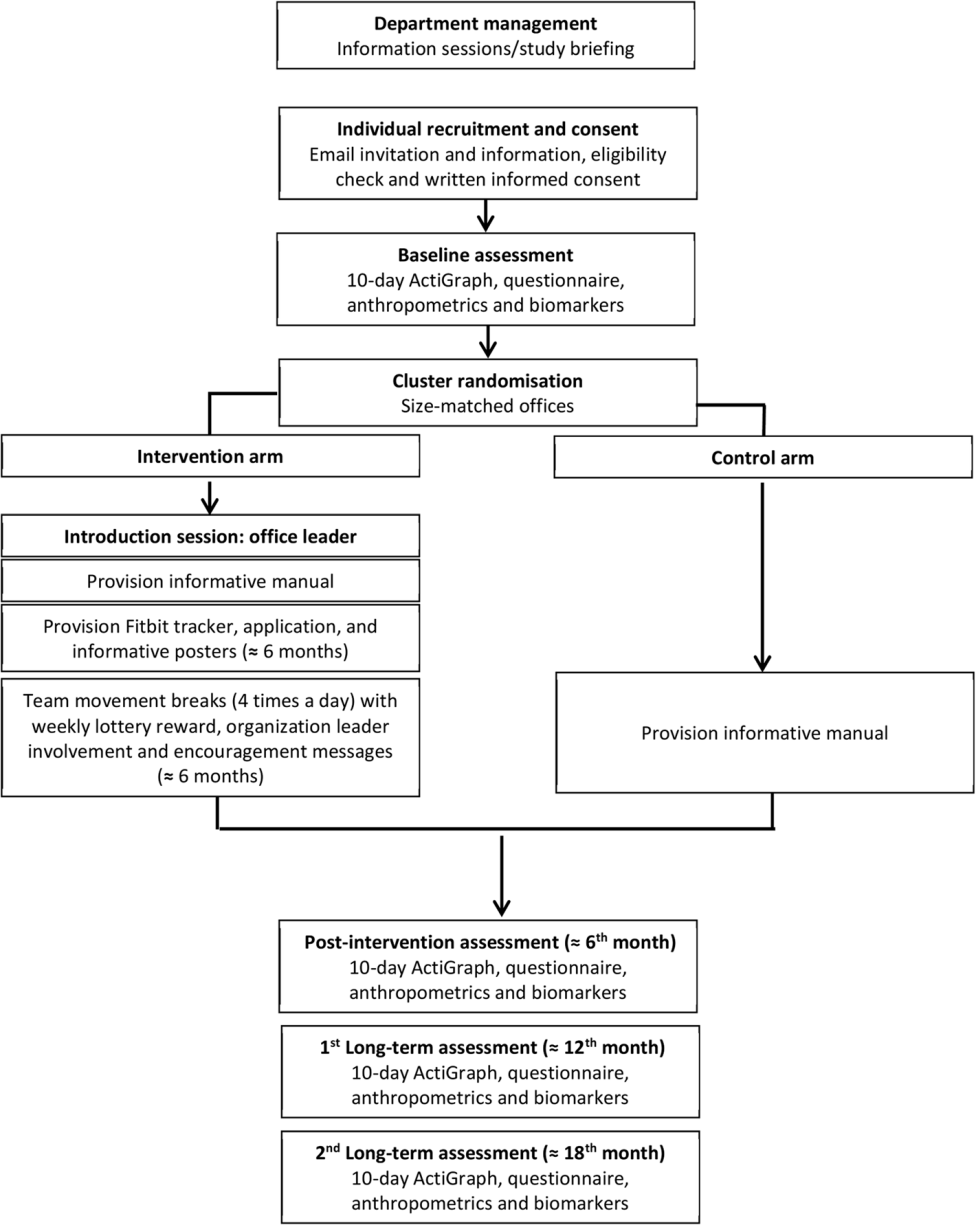
Study Overview

The duration of the research will be 18-months from the start of the intervention (6-months of intervention and 12-months follow-up, in addition to the baseline data collection), and participants will be requested to remain continuously enrolled during this period. Figure 1 provides an overview of the timeline of the PAW trial. Survey data will be collected at four time points: baseline, 6th month, 12th month and the 18th month after baseline assessment. Data will be collected by a trained research team, supervised by the core research team to ensure the quality and consistency of the data collected. The survey will be interviewer-administered. Trained physicians or nurses will take invasive cardiometabolic measurements at baseline, 6th month, and 12th month. Demographic information will only be measured at baseline. The main challenge will be to trigger sustained adherence to short breaks and not just a short-term behaviour change. In that regard, implementing a follow-up period that is sufficiently long to assess these long-term effects is essential. As a token of appreciation, all participants who have completed follow-up at 6th, 12th and 18th month will receive compensation of 500 THB (US $16) at 6th month, and 250 THB (US$ 8) each at 12th and 18th month. In total, they will be compensated 1000 THB (US $32) if they have participated until the end of the trial.

#### Primary outcomes

The primary outcome of this study is the change in sedentary time at 6th month follow-up. The accelerometer that will be used to measure SB is the ActiGraphTM wGT3X-BT tri-axial accelerometer (ActiGraph, Pensacola, Florida, USA). Participants will be advised to wear the ActiGraph accelerometer on their waist, above the right hip, with an elastic belt during their waking hours, excluding time spent in water-based activities, for a period of 10 days [39]. The device will be initialised at a sample rate of 60 Hz. Participants will be taught how to wear the device and will receive an information booklet with the instructions for reference. ActiGraph is a tri-axial accelerometer that measures SB and physical activities by categorising count data into different categories. These categories are defined as sedentary (150 and below counts per minute), light activity (151 to 2689 counts per minute), moderate activity (2690 to 6167 counts per minute) and vigorous activity (6168 and above counts per minute) [40]. Participants will be asked to wear the accelerometers during waking hours. Participants will be given activity logs to record the start and end date and time accelerometer wear. In addition, they will be asked to record their daily sleep and wake times as well as any activities, their frequency and duration, that they engaged in while not wearing the accelerometer (e.g. duration and frequency of water-based activities when they have to take off their accelerometer). The activity log will be used together with daily data to assess the adherence of wearing the ActiGraph accelerometers. To encourage adherence to wearing ActiGraph at night, participants will be given an additional 150 THB (US$ 5) per data collection. No incentive will be given if participants wear ActiGraph during sleep but not during the day. Participants will be able to receive an additional of 600 THB (US $20) in total if they participate in all data collection rounds, and wore ActiGraph both during the day and during sleep. Reminders to wear the accelerometers will be sent via the LINE messaging app.

#### Secondary outcomes

Secondary outcomes will include the impact of the intervention on PA such as time spent in light activities, and moderate to vigorous activities. The secondary PA outcomes will be measured using ActiGraph and the General Physical Activity Questionnaire (GPAQ) [41]. Other secondary outcomes include the change in on cardiometabolic outcomes. The cardiometabolic outcomes consist of the following physical measurements: waist circumference and neck circumference

- systolic blood pressure and diastolic blood pressure
- resting heart rate
- body weight
- height and body mass index (BMI).

Weight and height will be measured without shoes to the nearest 100 g and 0.1 cm, respectively. Body mass index (BMI) will be calculated by dividing weight in kilograms with the square of height in meters. Systolic and diastolic blood pressures will be measured after the participants had rested for at least 5 min [42] with an automatic blood pressure monitoring by trained research assistants. Waist circumference (cm) will be measured at the middle point between the lowest rib and iliac crest in the standing position. Neck circumference (cm) will be measured with the head positioned in Frankfurt horizontal plane using non-stretchable plastic tape to the nearest 1 mm. It will be measured at the level just below the laryngeal prominence perpendicular to the long axis of the neck.

Blood collection and tests will be done by a certified, private laboratory team (NHEALTH) [43] which consists of nurses and laboratory technicians. 15 ml of blood will be collected in each data collection. HOMA-IR will be calculated by [Fasting insulin (mU/l) x fasting glucose (mmol/l)]/22.5. Participants will be required to fast for at least 10 h before the blood tests. We will also be collecting blood biomarkers outcomes:

- fasting plasma glucose (HbA_1_c)
- fasting insulin
- homeostasis model assessment of insulin resistance (HOMA-IR)
- serum uric acid
- lipid profile
- high sensitivity C-reactive protein (hs-CRP).

Study participants with abnormal biomarkers will be encouraged to see physicians using their own public health insurances under the UHC policy which cover non-communicable disease treatments for all Thai population.

#### Tertiary outcomes

Quality-Adjusted Life Years (QALYs) will be obtained from three surveys for cost-effective evaluation. One is the EuroQol-5 Dimension (EQ-5D-5L), where participants will be able to rate the severity of their disease [44]. The instrument encompasses five dimensions: mobility, self-care, usual activities, pain/discomfort and anxiety/depression. Participants will be asked to rate their health state on five levels of severity. QALYs will also be taken from work productivity, which will be assessed using the Work Productivity and Activity Impairment Questionnaire (WPAI) and musculoskeletal health which will be assessed using the Standardised Nordic Questionnaire [45]. In addition, the study will ask participants for demographic information and self-report of comorbidities. For candidates with pre-existing health conditions or metabolic risk factors (e.g. hypertension, diabetes, hyperlipidemia) we will collect additional data on the history of disease and medication dosage.

#### Measurement timeline of outcomes

The primary outcome will be measured at baseline, 6th month, 12th month and 18th month follow-up. The non-invasive cardiometabolic outcomes will be measured at baseline and every 6 months until the 18th month. The invasive cardiometabolic outcomes will only be measured at baseline, 6th month and the 12th month. The tertiary outcomes such as the EQ-5D-5L, musculoskeletal health, WPAI and self-report comorbidities will be measured together with the non-invasive cardiometabolic measures at baseline, 6th, 12th and the 18th month (Table 1). The economic impact will be evaluated at 6th month and the end of the trial follow-up period.

**Table 1:** Overview of study outcomes and measurement instruments used (docx)

	Baseline	6 months	12 months	18 months
Objectively measured sitting, upright time and step count				
10-day ActiGraph	x	x	x	x
Self-reported demographics				
- Age	x			
- Gender	x			
- Nationality	x			
- Marital status	x			
- Education	x			
- Pre-existing diseases	x	x	x	x
- Smoking and alcohol consumption	x	x	x	x
Questionnaire:				
- Work Productivity and Activity Impairment questionnaire (WPAI)	x	x	x	x
- General Physical Activity Questionnaire (GPAQ)	x	x	x	x
- EuroQol-5 Dimension (EQ-5D-5L)	x	x	x	x
- Standardised Nordic Questionnaire	x	x	x	x
- Health utilisation	x	x	x	x
Health-related outcomes				
- Body weight	x	x	x	x
- Body height	x	x	x	x
- Neck, waist, and hip circumference	x	x	x	x
- Blood pressure	x	x	x	x
Biomarkers				
- Fasting plasma glucose, HbA1c	x	x	x	
- Fasting insulin	x	x	x	
- Lipid profile: triglyceride, total cholesterol, high density lipoprotein cholesterol (HDL-C), low density lipoprotein cholesterol (LDL-C)	x	x	x	
- High sensitivity C-reactive protein (hs-CRP)	x	x	x	

### Method monitoring

#### Data monitoring

There will be no data monitoring board. The study team will comply with data collection and management procedures which has been approved by the Thailand ethics committee and abide by the rules of medical confidentiality.

#### Harms

We do not expect adverse events to occur and will not collect data on adverse events as this will create an unnecessary burden on the participants. The short break leaders will remind participants to be careful when doing the movements and ask participants to check that there are no tripping hazards before they engage in any movements.

#### Auditing

There will be no independent audit of the trial. NHEALTH [43] and the data collectors from the MOPH will manage the security and quality according to their standard operating procedure.

#### Pilot feasibility study

We conducted a quantitative and qualitative internal pilot feasibility study with 20 convenience-sampled office workers (10 in intervention and 10 in control group) from a department, the Health Intervention and Technology Assessment (HITAP), in the MOPH. This study was conducted before the main study with two key objectives. Firstly, to assess the feasibility and acceptability of the interventions (e.g. having four short-breaks per day) and the criteria used to measure intervention adherence. Participant inclusion criteria were the same as for the main study. Two out of three offices from HITAP were chosen to take part in the study. The offices that were chosen were the furthest apart from one another to reduce the chance of spillover effects. Participants within the offices volunteered to take part in the study and were not randomly chosen. All participants wore the AcitGraph activity accelerometer to measure SB and PA. The intervention group also wore Fitbit devices to measure their step count during the short breaks. Findings from this pilot study informed iterations of the main study interventions and protocol. Secondly, this study included two interviews that were done to investigate the relevance of the interventions by understanding: (i) perceived definitions and knowledge related to SB and PA; (ii) perceptions related to SB and PA in the office including barriers and facilitators; (iii) opportunities to reduce SB and increase PA in the office; and (iv) how participants perceived the intervention and all its components. The results of the pilot study will be published separately.

#### Sample size

Eligible office workers from the DMS will be invited to participate in the trial. To detect a difference in the primary outcome of 23.3 min in sedentary time per day as reported in a prior randomised trial [22], the sample size was calculated with a standard deviation (SD) of 45.5, based on a two-tailed significance level of 5% and power of 80%. The calculation assumes a conservative intra-cluster correlation coefficient of 0.05 and a coefficient of variation of 0.52 to account for varying cluster sizes in the study. There are 18 clusters (offices), each cluster varying between 7 to 40 office workers. The mean cluster size was 22.1 with a standard deviation of 11.6. Assuming an average cluster size of 22 participants, we will yield a design effect of 2.36. Therefore, the total sample size required to detect a difference of 23.3 min in SB is 288 participants. To conform to best practice, this sample size will be inflated by 20% to account for dropouts and potential loss of participants due to non-compliance to primary outcome or chances of faulty devices. Hence, a sample size of 360 participants is needed.

### Data analyses

#### Primary and secondary outcomes

The primary aim of this study is to investigate the impact of introducing short movement breaks during office hours on SB of office workers both during and outside working hours. This primary analysis will be conducted using a linear mixed-effect model with daily time spent in SB as the dependent variable and the intervention allocation as the independent variable. The model will be adjusted for confounders such as baseline SB, baseline characteristics (such as age and gender), device wear times and stratification factor defined by office size, when necessary. The model will account for possible intra-cluster correlation of individuals within the same office.

In our primary analysis mentioned above, we will adopt the complete case analysis approach where missing values will not be replaced. Secondary and tertiary outcomes relating to PA, cardiometabolic outcomes, quality of life, and productivity will be analysed using a similar methodology. These analyses will be conducted at 6-month and 12-month follow-up to investigate the effectiveness of the intervention as well as post-intervention behavioural maintenance. Sensitivity analysis using the intention-to-treat approach where missing data will be imputed will also be carried out for the primary outcome of our study. Baseline characteristics of participants who are lost to follow up or dropped out of the study will be compared to those who completed the study to investigate differential dropout between intervention and control groups. All evaluations will be performed using a two-sided test at 5% level of significance.

#### Economic evaluation

The economic evaluation aims to report two pieces of cost-effectiveness information of the PAW program: 1) short-term economic impact; and 2) long-term economic impact. The analysis will be conducted from the perspective of public health care payer. The costing data will be collected from the trial. The longer-term costs, e.g. treatment costs for stroke and diabetes, will be retrieved from domestic literature or relevant cost databases [46].

First, we aim to conduct a person-level economic evaluation to report a short-term economic impact of the PAW program using the data from the trial within the study period. Based on data from the trial, we will compare the costs and outcomes of the intervention against the control using the net benefit regression framework [38]. Costs will include costs incurred to the MOPH, including intervention costs and costs of treating related health problems (e.g., musculoskeletal diseases, diabetes and hypertension). The effectiveness outcomes for this analysis will include: 1) absolute working hours; 2) absenteeism and presenteeism; 3) key clinical indicators, e.g. cardiovascular disease (CVD) risk scores; and 4) QALYs. Separate analyses will be conducted for each outcome. The output of this analysis will be expressed as an incremental net benefit of PAW compared to no PAW. We will also estimate incremental cost-effectiveness ratios (ICERs) over the study period, for example, the incremental cost of the PAW program (compared to no PAW) to obtain one more absolute working hour, and incremental cost for one more QALYs gained. The use of regression will allow us to adjust for potential confounders and to calculate the clustered standard errors using the sandwich variance estimators of Huber and White [39] to account for a clustered randomised design in which the outcomes of interest may be correlated. Uncertainty of the cost-effectiveness findings within the study timeframe will be characterised using a cost-effectiveness acceptability curve and a 95% confidence interval [40]. Moreover, additional subgroup analyses (e.g., by sex and age groups) could be explored.

**Fig. 2:**
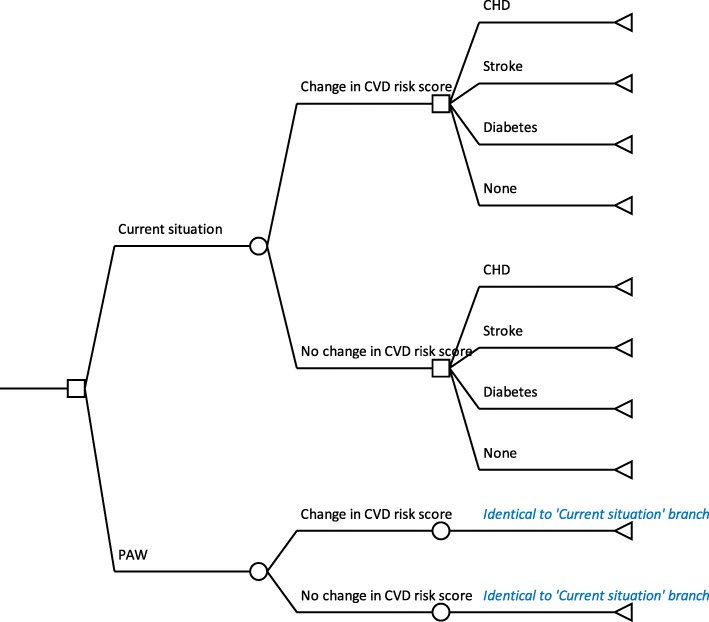
The Decision tree and Markov model for assessing costs and outcomes of PAW compared to the current situation (no PAW implemented)

Furthermore, we aim to construct a hybrid model, with a decision tree and Markov models, also using the public health care payer’s perspective. Model-based economic evaluations will help to assess the economic impact and benefits of PAW implemented at the workplace in the long term. Decision tree model (Fig. 2) compares the current situation where PAW has not been implemented and the alternative policy option of PAW. The outcome of each choice will be whether or not a change in cardiovacular disease (CVD) risk scores (based on Rama-EGAT heart score) [47, 48] occur after exposing to the interventions. Next, the Markov model will be performed to predict lifetime costs and outcomes that occurred after the change in CVD risk scores. The Markov model, which will run separately using transitional probabilities, follows the natural history of coronary heart disease (CHD), stroke, and diabetes based on the CVD risk scores. The Markov model will be run using the lifetime time horizon with a cycle length of 1 year. All costs and outcomes occurring after 1 year will be discounted at a rate of 3%, as recommended by iDSI Reference Case [49]. Inputs to the models will be obtained from the trial and literature review. The outputs from cost-effectiveness analysis will be presented as cost, QALYs, and ICER to reflect value for money of PAW. Sensitivity analyses, including univariate and probabilistic sensitivity analysis, will be performed to describe the uncertainty of the findings.

Lastly, the budget impact analysis of PAW will be conducted to estimate the 5- and 10-year financial implication of adopting PAW at the workplace to public health care payer.

## Discussion

This paper describes the design of a cluster randomised trial that will evaluate the effects of the multicomponent PAW behavioural intervention on SB and other outcomes in desk-based office workers in a low- and middle-income country. This type of studies in a given setting is very rare in the current literature. The PAW intervention builds on the previous effort of the MOPH, and previous experiences of the research team in behavioural health interventions focused on SB and PA, as well as movement behavior monitoring [50–53]. Others have shown that activity accelerometers, together with financial incentives, are promising environmental intervention to reduce occupational sitting time and increasing PA at least in the short-term [54] and possibly also in the longer term.

This evaluation will identify changes in key movement behaviours via Fitbit real-time data, as well as objective measures via ActiGraph on time spent in sedentary, light, moderate or vigorous activities as well as step counts, intermediate health outcomes as well as work-related outcomes. We will also include policy-relevant economic evaluation through both a Markov model and person-level cost-effectiveness analysis to report a short-term health and economic impact of the PAW program using the data from the trial within the study period. These will be used to formulate recommendations for future improvement and refinement of the intervention, which will be essential in the light of the potential wider implementation and roll out by the MOPH.

### Potential difficulties and limitations and alternative approaches

Identification of effect sizes might be indirect for data with repeated measures. As such, care will be taken to use alternative estimation techniques such as multilevel linear mixed-effect model and adjusted standard errors to ensure robust results from treatment effects when evaluating the impact of the interventions. We may not be able to follow people for a prolonged period due to loss to follow-up.

In conclusion, the current cluster randomised controlled trial will assess the effects of a multicomponent PAW behavioural intervention in reducing sitting time and increasing PA in desk-based office workers in the longer-term as compared to usual practice. In addition, from a company and societal perspective, we will provide insight into the cost-effectiveness of the intervention as compared to usual practice. We will also assess if a reduction in sitting time and increase in PA is related to the quality of life, health and work-related outcomes, and how the PAW intervention can be further improved.

## Supplementary information

**Additional file 1.** Overview of BCTs and SEM levels used in the PAW study. Table of Behaviour Change Techniques (BCTs) and Socio-ecological model (SEM) used in the PAW study.

**Additional file 2.** SPIRIT checklist. SPIRIT checklist 2013 PAW study.

**Additional file 3.** Poster of exercises and stretching. Images of three posters used in the PAW study.

## Data Availability

The research team will have exclusive rights to the de-identified data for 24 months after the trial is completed. After that, the data and full protocol will be publicly accessible on the HITAP website.

## Abbreviations

API: Application programming interface
BCTs: Behaviour change techniques
BMI: Body mass index
CHD: Coronary heart disease
CM: Centimetre
CVD: Cardiovascular disease
DMS: Department of Medical Services
ECMOPH: Ethical Review Committee for Research in Human Subjects, Ministry of Public Ministry
GPAQ: General Physical Activity Questionnaire
HbA_1_c: Fasting plasma glucose
HDL-C: High density lipoprotein cholesterol
HITAP: Health Intervention and Technology Assessment
HOMA-IR: Homeostasis model assessment of insulin resistance
hs-CRP: High sensitivity C-reactive protein
ICERs: Incremental cost-effectiveness ratios
ICMJE: Committee of Medical Journal Editing
LDL-C: Low density lipoprotein cholesterol
METs: Metabolic equivalents
MOPH: Ministry of Public Health, Thailand
PA: Physical activity
PAW: Physical Activity at Work
SB: Sedentary behavior
TCTR: Thai Clinical Trials Registry
THB: Thai Baht
UHC: Universal Health Coverage
US: United States
