# Supplementary material for: The physical activity at work (PAW) study protocol: a cluster randomised trial of a multicomponent short-break intervention to reduce sitting time and increase physical activity among office workers in Thailand
Source: BMC Public Health. 2020 Sep 1;20:1332. doi: 10.1186/s12889-020-09427-5 (PMC7466487; doi:10.1186/s12889-020-09427-5)
Supplement: Supplementary file 1 — Additional file 1. Overview of BCTs and SEM levels used in the PAW study. Table of Behaviour Change Techniques (BCTs) and Socio-ecological model (SEM) used in the PAW study. [file 12889_2020_9427_MOESM1_ESM.docx]

| **Table 1a: Overview of BCTs and SEM levels used in the PAW study** | | | |
| --- | --- | --- | --- |
| **Intervention Component** | **Behaviour Change Technique** | **Definition of Behaviour Change Technique** | **Implementation** |
| **Individual Components** | | | |
| Lottery-based incentives | ***Material incentive (behaviour)***  ***Material reward (behaviour)*** | Inform that money, vouchers or other valued objects/verbal or non-verbal reward ***will be*** delivered if and only if there has been effort and/or progress in performing the behavior  Arrange for the delivery of money, vouchers or other valued objects/verbal or non-verbal reward if and only if there ***has been*** effort and/or progress in performing the behaviour | Participants will be informed that they have the chance to win 500 THB (US$ 16) if they participate in 60% of short breaks per week. A lottery will decide the selection of the winner among eligible participants. |
| Activity device – Fitbit Inspire HR | ***Feedback on behaviour*** | Monitor and provide informative or evaluative feedback on performance of the behaviour (*e.g. form, frequency, duration, intensity)* | Fitbit Inspire HR device together with the Fitbit app allows participants to see, amongst other outcomes:  -steps taken for the day  -calories burned  -distance covered  -active minutes  -heart rate |
| Information booklet | ***Information about health consequences*** | Provide information (e.g. written, verbal, visual) about health consequences of performing the behaviour | An Information booklet about the health consequences of PA and reducing SB will be given to participants at the start of the intervention. The booklet will be given to both the intervention and the control group. |
| **Physical Component** | | | |
| Posters | ***Information about health consequences***  ***Instruction on how to perform behavior***  ***Behaviour substitution***  ***Material incentive (behaviour)*** | Provide information (e.g. written, verbal, visual) about health consequences of performing the behaviour  Advise or agree on how to perform the behaviour  Prompt substitution of the unwanted behaviour with a wanted or neutral behaviour  Inform that money, vouchers or other valued objects ***will be*** delivered if and only if there has been effort and/or progress in performing the behaviour | Posters with information about the health consequences of SB and PA, ways to replace SB in the office and information about the study (e.g. rewards) will be displayed in the offices of the intervention participants.  **Appendix Figures 1a-c** |
| **Cultural Component** | | | |
| Team movement breaks | ***Behaviour substitution***  **Prompts/cues**  ***Action Planning***  ***Habit formation***  ***Habit reversal***  ***Instruction on how to perform a behaviour***  ***Demonstration of the behaviour***  ***Social comparison*** | Prompt substitution of the unwanted behaviour with a wanted or neutral behaviour  Introduce or define environmental or social stimulus with the purpose of prompting or cueing the behavior. The prompt or cue would normally occur at the time or place of performance Note: when a stimulus is linked to a specific action in an if-then plan including one or more of frequency, duration or intensity also code 1.4, Action planning.  Prompt detailed planning of performance of the behavior (must include at least one of context, frequency, duration and intensity). Context may be environmental (physical or social) or internal (physical, emotional or cognitive) (includes *‘***Implementation Intentions***’*)  Prompt rehearsal and repetition of the behaviour in the same context repeatedly so that the context elicits the behaviour  Prompt rehearsal and repetition of an alternative behavior to **replace** an unwanted habitual behavior  Advise or agree on how to perform the behaviour  Provide an observable sample of the performance of the behaviour, directly in person or indirectly e.g. via film, pictures, for the person to aspire to or imitate  Draw attention to others’ performance to allow comparison with the person’s own performance Note: being in a group setting does not necessarily mean that social comparison is actually taking place | An alarm and short break leader will prompt participants to take the short movement break, at the same time and place each day where feasible  Participants will be instructed to take 4 breaks that are 3-5 minutes in length per day.  An exercise or dance video with music will be played and participants are encouraged to dance/exercise along with the video instructor. |
| **Organisational Component** | | | |
| Leadership support | ***Information about others’ approval*** | Provide information about what other people think about the behaviour. The information clarifies whether others will like, approve or disapprove of what the person is doing or will do | DMS leaders will use LINE, a messaging app in Thailand, to convey their approval of the study and encourage the intervention participants to take part in the interventions. |
| Social incentive and reward | ***Social incentive***  ***Social reward***  ***Social comparison*** | Inform that a verbal or non-verbal reward ***will be*** delivered if and only if there has been effort and/or progress in performing the behavior (includes ‘**Positive reinforcement’**)  Arrange verbal or non-verbal reward if and only if there ***has been*** effort and/or progress in performing the behavior (includes ‘**Positive reinforcement**’)  Draw attention to others’ performance to allow comparison with the person’s own performance Note: being in a group setting does not necessarily mean that social comparison is actually taking place | The reward will be handed out to the winner by a DMS leader and the participant will be praised for performing well.  Participants will be told about the reward-lottery in advance in the information booklet. |

**References**

1. Mullane SL, Toledo MJL, Rydell SA, Feltes LH, Vuong B, Crespo NC, et al. Social ecological correlates of workplace sedentary behavior. Int J Behav Nutr Phys Act. 2017;14;1.

2. Michie S, Richardson M, Johnston M, Abraham C, Francis J, Hardeman W, et al. The behavior change technique taxonomy (v1) of 93 hierarchically clustered techniques: building an international consensus for the reporting of behavior change interventions. Ann Behav Med. 2013;46;1:81-95.
