# Supplementary material for: The physical activity at work (PAW) study protocol: a cluster randomised trial of a multicomponent short-break intervention to reduce sitting time and increase physical activity among office workers in Thailand
Source: BMC Public Health. 2020 Sep 1;20:1332. doi: 10.1186/s12889-020-09427-5 (PMC7466487; doi:10.1186/s12889-020-09427-5)

**Figure 1a: Poster of exercises and stretching**


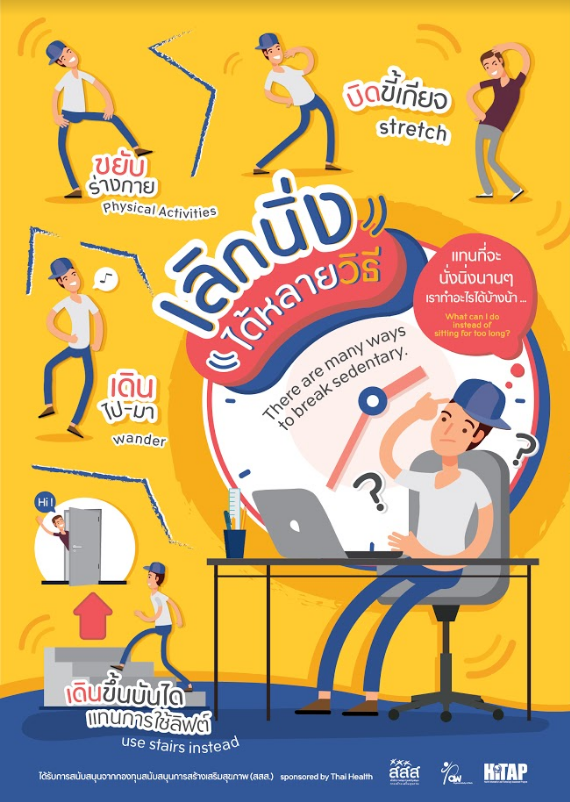


**Figure 1b: Poster of exercises and stretching**


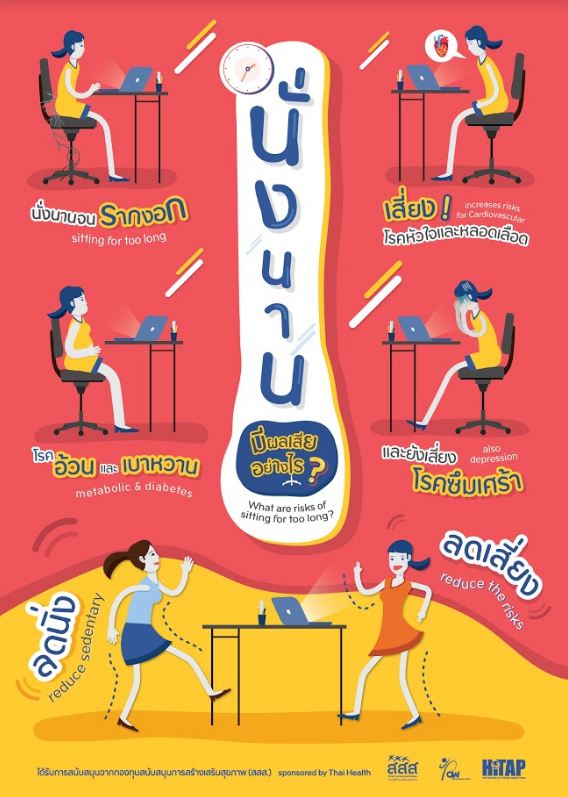


**Figure 1c: Poster of exercises and stretching**


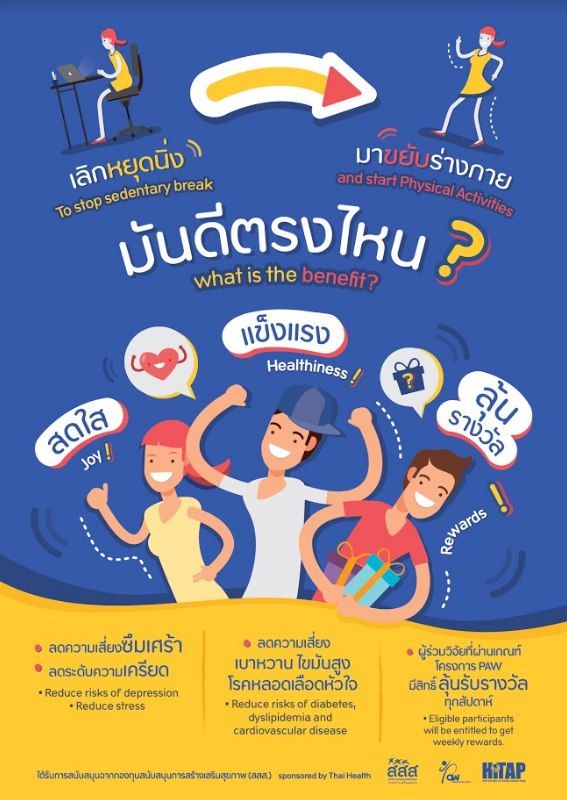

Supplement: Supplementary file 3 — Additional file 3. Poster of exercises and stretching. Images of three posters used in the PAW study. [file 12889_2020_9427_MOESM3_ESM.docx]
